# Health Hybrid Concept Analysis in Old People

**DOI:** 10.5539/gjhs.v5n6p227

**Published:** 2013-10-31

**Authors:** Ahamadali Asadi Noghabi, Fatemeh Alhani, Hamid Peyrovi

**Affiliations:** 1 School of Medical Science, Department of Nursing, Tarbiat Modares University, Tehran, Iran; 2 School of Nursing & Midwifery, Tehran University of Medical Sciences, Tehran, Iran

**Keywords:** concept analysis, elderly health, hybrid approach

## Abstract

**Background:**

It seems necessary to study the health status of this age group to promote their health and prevent disease as well as care planning. In order to achieve this goal, a clear definition of the concept of elderly health is essential.

**Method:**

Hybrid concept analysis, our research design, utilizes both theoretical analysis of literature and empirical observation to define a concept. We chose the hybrid concept analysis method because its inclusion of old people perspectives enriches the limited health research literature. The method consists of three phases

theory, fieldwork, and analysis.

**Results:**

In comparison, we can conclude that health in the elderly people is something more than the absence of illness and 4 physical, mental, social and spiritual domains which are referred to in the definition of a theoretical stage are supported by the findings. The relative health was also proposed against the complete welfare and comfort for the elderly and it showed that their expectations are less than their ages. In addition, the elderly have expressed the family as a preference and the researcher believes that this theme is context based because it has emerged following the interview. Since the family has a special place according to the Iranian culture and religion and the family health is a priority in their health. In addition, the daily activities have been raised as a major theme that can be considered as the physical health but the elderly have expressed it apart from the physical health.

**Conclusion:**

Health among the old is a concept that is affected by genetic, environmental, healthcare services and lifestyle-related factors and involves proportional physical, mental, social, familial, spiritual, and economical welfare along with the ability to handle daily life activities which is measurable through medical and functional approaches.

## 1. Introduction

The old population is probably the most important demographic phenomenon of the world in the late 20^th^ century and the early 21^st^ century. Old age is attributed to the individuals who are above 65 years and currently, it has a growth rate of 2.4 percent compared with 1.7 percent of total population. Growth rate is expected to reach 3.1 percent in coming years. 2025 Generally speaking, the old population will increase from 600 million in 2000 to 1 billion and 200 million, i.e. twofold in (Ocampo, 2010).

Aging process can lead to mental and physical attrition and a reduction of active and healthy life expectancy. Changes in well being often get more chronic and progressive while age increases and is less returnable (Ocampo & Landono, 2007).

With regard to the above-mentioned points, it is necessary to consider the health condition of this age group in order to promote their health and prevent diseases while taking appropriate measures for taking care of them.

In 1945, the World Health Organization defined health as a physical, mental and social sense of wellbeing and having no diseases or disabilities (Rob, 2000). This definition was later described in four physical, mental, social and spiritual areas and also emphasized on looking at human as a total and one entity.

As a result of such a holistic perspective, examining the health among the old is a complex task since a series of internal and external balances occur during aging, including changes in the function of individuals’ organs. These changes make individuals less adaptable when they face stress which ultimately could lead to defects in their organs and diseases (Eliopoulos, 2010).

In order to investigate health among the old, it is mandatory to consider psychological, familial, social, economic and functional areas. Such an investigation must incorporate more the mental aspect compared with other age groups since it is dependent on interaction through physiological situations, sense of psychological health, functional abilities and social supports. For this reason, investigating the old people’s health must not be only limited to medical aspects and it must get in touch with clinical specialists, decision makers and the researchers who work with this age group (Walker & Avant, 2005). Objective literature on hybrid concept analysis is to create the foundation for the next step in deeper analysis and refinement of the concept.

## 2. Method

Hybrid concept analysis, our research design, utilizes both theoretical review of literature and empirical observation to define a concept (Fowler, 1998). We chose the hybrid concept analysis method because its inclusion of old people perspectives enriches the limited health research literature. The method consists of three phases: theory, fieldwork, and analysis. The theoretical phase initiates the research process with a review of the literature. Its purpose is to capture the overall essence of a concept and how it is defined, used, and measured in the literatures (Webster, 2011). This phase results in the selection of a working definition of the concept. The fieldwork phase further refines the concept definition by collecting qualitative data from patients. In the analytic phase, data from the fieldwork phase are compared with findings from the theoretical phase to produce a refined definition of the concept that is supported by both literature and patient perspectives (Husseini, 2010; WHO, 2009).

### 2.1 Theoretical analysis of concept

The theoretical stage starts with literature review and aims at reaching to the total nature of the concept and the way of defining and measuring it in studies. The results of this stage are applied in choosing the practical definition of concept at field stage and correcting the gained concept from qualitative data about the old (Shanas, 1971).

#### 2.1.1 Concept Selection

A concept for a study is selected differently in nursing research. With regard to this point that this study is a part of the main research called to design the instrument for examining the condition of health among the old and conducting its psychoanalysis, the analyzed concept is the old people’s health.

#### 2.1.2 Search of Studies

Search of studies starts in this stage and then is developed and kept up with at field stage. Finally, the aim of this stage is a comprehensive review over the studies about the intended concept and reaching to a deep understanding in different fields of science at any time. Considering the aforementioned points, the review over studies was carried out by using the databases of CINAHL, SCOPUS, PUBMED, PROQUEST, SCIENCE, ISC, IRANDOC, MAGIRAN by searching the phrases of Health, the Elderly Health, Health Status, Health Measurement, Health Assessment besides the expressions of Elderly People and Old Age without time limitation.

The researcher started the review of studies by the following qquestions: How has health being defined? How had the old people’s health been conceptualized? How has the old people’s health been measured?

Throughout the review over the studies, 741 titles were obtained in initial search which were condensed into 69 after studying the titles. Afterwards, the abstracts of papers were reviewed and 34 papers, which were congruent with the goals of research, were slelcted. In addiion, 4 reference books in the fields of the old people nursing, the old people’s health and measuring instruments were gone over and accordingly, three eldelry theories which were related to health were picked out.

**Figure 1:**
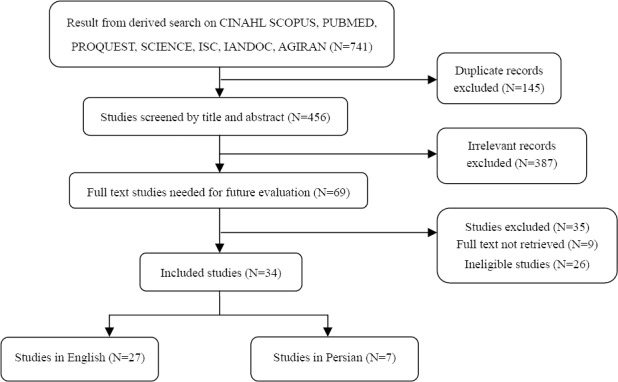
Flow chart of search process

Studies showed that there were two main approaches for dientifying the health-releated needs which are called Medical and Functional Models. Individuals who believe in the medical model maintian that physical examinations are necessary for pinpointing the the old people’s health and their health-related needs and another group beilieved that the issues expressed by an old person could be the best index for their health condition and their required supportive systems (Damian, Pastor, & Martín-Moreno, 1999). Investigating the old people’s health with the medical approach has three stages of getting record, physical examinaitons and laboratorial along with diagnostic tests.

The functioning condition means the ability of individual to carry out necessary activities, to have a sense of wellbeing and integrity in three areas of biological, psychological (cognitive and emotional) and social functioning (Liang, Krause, Bennett, Kobayashi, & Fukaya, 2005).

The comprehensive investigation of the old has high significance due to the prevalence of chronic diseases and the increase of the risk for having functional disabilities in this life period. The key points that must be considered while investigating them are as follows:

1)- most of the old have more than one health or social problem.
2)- investigating a problem usually can lead to the discovery of its related problems.
3)- the reason or the mechanism of a problem can be better understood if the source of problems that have emerged with increase of age is considered.
4)- forcasting of the intial problem could be different from that of the problems related to it.
5)- appropriate therapy is dependent on all problems and it might be necessary to control all problems and then prioritize them.
6)- it is quite possible that the improvement of a problem lead to the deterioration of another problem.

Different researches have indicated that measuring the perceived health of the old was a universal and useful index for their health level and was interactional with its biological and social factors (Wong & Palloni, 2005). As a matter of fact, the percieved health is one of the most common types for the identification of health condition among the old from a cognitive perspective since it could examine different aspects of health. For instance, objective measurement often assesses one area like identifying the level of glycolized hemoglobin which is used for controlling the meliutus diabetics. As a result, the perceived health attends to different apsects of health that are recommended by the World Health Organization (Borrell-Carrio & Epstein, 2004). Considering the third question which was in the researcher’s mind for analyzing the concept of theroy, the studies that measured health were reviewed.

First of all, we must see what is to be measured. In health nursing and researches, these issues are usually attended to: measuring cognition, emotion, psychological and kinesic performance and physical performance (Albala et al., 2005).

#### 2.1.3 Dealing with Meanings

While we have a few definitions available, searching the prominent issues that are similar and contradictory in these meanings at this stage could be useful.

In analyzing different tests relevant to the old people’s health, it was found that the most comprehensive definition of health was the sense of physical, mental, social and spiritual wellbeing besides having no diseases or disabilities.

The factors that are effective on health can be categorized into four groups which include the genetic factors (10%), lifestyle (50%), health services (20%) and environmental factors (20%) (Walker & Avant, 2005). The effective factors on the the old people’s health are categorized into six main groups of demographic (gender, age, level of education, income, social security), social variables (family and caregivers’ performance), biological variables (chronic diseases, medicine, cigar and alcohol consumption), mental variables (alzheimer disease, depression), functional variables (doing daily life activities, speed of wlaking, level of energy) and the syndroms related to old age (collapse, lack of control over urination). Althimer.

The prevalence of chronic disease and the risk of functional disabilities have led to specialized recommendations in examining the health of the the old: most of the old people have more than one health or social problem,

#### 2.1.4 Workable Definition

The last stage of the theoretical analysis of concept within the hybrid model is presenting a workable definition.

Health among the old is concept which is affected by genetic factors, environment, health care services and lifestyle and its important dimensions are physicl, mental, social and spiritual which are measurable through medical and functional approaches.

### 2.2 Empirical Concept Analysis

The aim of in-field phase is the incorporation and identification of a concept by analytical development and completion which had begun at phase one and it usually starts before the theoretical phase finishes. The aim of this phase is strengthening and modifying a concept by developing and completing the analysis which had begun at the first phase and is carried out via experimental observations. This stage includes the fundamental steps that are common in qualitative research. This stage involves 4 steps: 1) preparing the condiitons, 2) examining the conditions and entering into the discussion over the topic, 3) selecting the samples and 4) collecting and analyzing the data (Schwartz & Kim, 1993).

#### 2.2.1 The Step of Preparing the Conditions

The emphasis of this step is on selecting the field setting, identifying the main questions for the manual at field stage, selecting the location of data collection and listing the locations in which the samples might exist in them. Considering that the stipulated concept in this study is the old, three criteria which act as guides for selecting the population and conditions are: 1) the high possibility of recurrent observations of the researched phenomenon, 2) the appropriateness of environment for collecting the data, 3) the possibility of the researcher to place the participants at observation setting.

One or two main questions that could be used for the intended concept are selected. The questions that guide the in-field phase must: 1) include the main compartments of the concept, 2) the components that differentiate the main concept from similar concepts, 3) measuring criteria that might develop the concept.

The questions that were identified for interviewing the old in previous phase were:

-How is the definition of health for you? How kind of feeling do you have about your own health?
-what kind of effect has increase of age had on your health?
-How can society help to promote your health?

#### 2.2.2 Samples Selection

Considering that the analyzed concept is the old people’s health, the samples were selected from among the individuals who were above 65 and higher. Since the number of samples was high, eight old men and women (regarding the effect of gender on health) were selected from among the different age groups of the eldelry and were accorsingly interviewed.

If the essential characteristics of concept are not clear and straightforward indicses are not found, the Wilson Method can be used for analyzing concept. This typology starts with introducing a sample. The sample can be an instance of a thorough reflection of concept. The second case is the contradictory case which is an instance of the one which is contradictory with cocnept. The third case is the boarder one which is an instance that reports an abnormal case and helps highlight the materials that resemble to the real case. Finally, this method ends up with relevant case and leads to the clarification of the central concept by identifying the criterion for a relevant case.

#### 2.2.3 Data Collection

Schwatzman and Struss (1973) consider the in-depth interview system as appropriate for data collection, recording and analysis for analyzing concepts. The method of data collection in this research method is semi-structured interveiw; the intterview starts with the questions that have been attained at theoretical concept analysis stage and then was kept up with regard to the responses of participants dursing interviews.

Data collection Method:

The data were collected from the old in this research by using semi-structured interviews.

#### 2.2.4 Analyzing the Field Data

The researcher enters the analysis stage at the same time as he collects the data. Data collection and analysis are carried out simultaneously. After going through half or three fourth of the in-field stage, the researcher needs to extract and organize the data that are relevant to concept.

Since qualitative study can come up with a new insight and the most appropriate approach for discovering the essences, meanings, temperaments and sensational manners, characteristics, and values is the life of individuals or the representative group (Ocampo, Saa, Herrera, & Reyes-Ortizand, 2006). We use a qualitative model in this research which was content analysis. Content analysis is used for interpreting the content of textual data (Lima-Costa & Uchoa, 2004).

The main themes resulting from content analysis:

Physical (biological), Mental (psychological), Family (emotional), Community (social), Activity daily living (functional area), Spiritual, Lifestyle

### 2.3 Final Analysis Stage

In this part, the findings of the theoretical content analysis stage are compared with the in-field findings. Regarding that the empirical definition that was obtained at theoretical stage included the fact that health among the old is a concept that is affected by genetic, environmental, caring services and lifestyle factors, it also implies important dimensions including physical, mental, social and spiritual dimensions which are measurable through medical and functional approaches. Investigating health among the old must be more mentally oriented compared with other age groups. The most important consequences of health promotion among the old are longevity, improving quality of life, sense of wellbeing and boosting of self confidence.

The main themes of the in-field stage are physicla health (biological), mental health (psychological), family health (emotional), cpmmunity health (social) and handling daily life activities (functional area), spiritual health and lifestyle.

On the other hand, it can be concluded that health among the old is more than lack of disease and the findings of the in-field stage support the four physical, mental, social and spiritual areas which are pointed out at the theoretical definition stage. The proportional nature of health was also mentioned along with complete tranquility and prosperity by the old which shows the low level of expectations among them by virtue of their age.

In addition, the the old have mentioned family health as one of their priorities. The researcher guesses that considering that this theme has emerged as an outcome of interviews, it can be claimed that it is context-centered and by virute of the Iranian people’s culture and religion in which family has a unique status, activity daily living has been mentioned as one of the main themes. Although it can be deemed as one of the subcategories of physical health, the old mentioned it as separate from physical health and they emphasized on lifestyle from among the factors that contribute to health.

## 3. Final Definition

Health among the old is a concept that is affected by genetic, environmental, healthcare services and lifestyle-related factors and involves proportional physical, mental, social, familial, spiritual, and economical welfare along with the ability to handle daily life activities which is measurable through medical and functional approaches.

Investigating health among the old must be more mentally-oriented in comparison with other age groups. The most important consequences of health promotion among the old are longevity, improving quality of life, sense of wellbeing and boosting of self confidence.
